# Human Follicle *in vitro* Culture Including Activation, Growth, and Maturation: A Review of Research Progress

**DOI:** 10.3389/fendo.2020.00548

**Published:** 2020-08-11

**Authors:** Qiyu Yang, Lixia Zhu, Lei Jin

**Affiliations:** Reproductive Medicine Center, Tongji Hospital, Tongji Medical College, Huazhong University of Science and Technology, Wuhan, China

**Keywords:** *in vitro* culture, human follicle, human oocyte, *in vitro* maturation, fertility preservation

## Abstract

Fertility preservation has received unprecedented attention nowadays. In addition to cryopreservation and re-implantation of embryos, oocytes, and ovarian tissue pieces, *in vitro* culture system for follicles/oocytes has been considered as an alternative strategy for fertility preservation. Since the metabolic dynamics and required nutrients are not entirely the same in different stages of follicular development, optimization of each culture step is needed. In this paper, literature regarding culture conditions in three steps were analyzed. Known additives in activation stage included 740Y-P, bpV(HOpic), follicle stimulating hormone (FSH), human serum albumin (HSA), ITS, growth differentiation factor 9 (GDF9), bone morphogenetic protein 15 (BMP15), and cyclic adenosine monophosphate (cAMP), with different degrees of activation promotion and potential detrimental effect on DNA integrity. For isolated follicles growth stage, actin A, FSH, basic fibroblast growth factor (bFGF), estradiol were proved to improve development or proliferation. As for maturation, addition of growth hormone, melatonin, C-type natriuretic peptide (CNP), GDF9, cilostamide, or forskolin helped to regulate maturation rate or improve oocyte quality. Based on previous sequential culture system for human follicles, optimization is needed to achieve higher maturation rate and better oocyte quality, pursuant to current review, which demonstrated the effects of various additives on different stages.

## Introduction

In recent decades, considerable progress has been made in the field of assisted reproductive technology (ART). Objectively, with improvement of the prognosis of malignant diseases and progress in ART, an increasing number of female cancer patients have the opportunity to preserve fertility through advanced technologies. Subjectively, young cancer patients are no longer satisfied simply with the continuation of life span, but become aware of their fertility needs. Therefore, fertility preservation has received unprecedented attention (1, 2). Thanks to current technology, part of the ovarian tissue of the women who need chemotherapy due to malignancies could be removed for cryopreservation before receiving treatment, so as to avoid the reproductive toxicity of chemotherapy drugs, and then be auto-transplanted at the end of treatment. However, the risk of re-implantation of tumor cells is faced by some patients during transplantation (3, 4). To solve this problem, an *in vitro* culture (IVC) system for follicles and oocytes has been considered as an alternative fertility preservation strategy without the risk of re-implanting maternal somatic tumor cells (5).

For most mammals, the formation of follicles takes place pre-natally and constitutes the basic reserve unit of female reproductive cells, which are recruited for growth throughout the female's reproductive age. Specifically, development of follicles includes (1) initiation of primordial follicle growth and development to the preantral follicle stage; (2) the formation of antral follicles; and (3) rupture of the Graafian follicle releasing a cumulus-oocyte complex (COC) at ovulation in response to the mid-cycle luteinizing hormone (LH) surge (6). The IVC system should support the development of oocytes in all stages, from activation of primordial follicle to oocyte maturation. Once the safety and effectiveness of the system are confirmed, the maximum use of the ovarian follicle reserve would be achieved, and the risk of tumor cells carried by transplanted ovarian tissue would be eliminated. Moreover, for younger patients like prepubertal girls, neither mature oocytes nor embryo cryopreservation are available. Therefore, fertility preservation may be achieved by the IVC system (3, 7, 8).

Most follicles are in the primitive stage, and their recruitment is regulated by both endocrine factors and the ovarian internal environment (9, 10). During *in vivo* follicle growth, oocytes acquire the competence to resume meiosis, complete fertilization, and support early embryonic development. Thorough comprehension to this complicated growth procedure is essential for researchers to construct an IVC system meeting all requirements, with consideration for maintaining DNA integrity and stability (11, 12). In previous studies, a two-step culture system was designed for follicular development in mouse models, where the IVC oocytes were fertilized and healthy offspring were successfully obtained (13, 14). Similarly, mature oocytes from IVC cattle follicles were fertilized and a healthy calf was born with higher birth weight (15). These results in animal models proved the feasibility of follicular IVC. However, the inconsistency of follicular growth environment between humans and animals and challenges of applying the system to human follicles still need to be considered (16). A single research reported the multi-step IVC system for the whole course of human oocyte development, and some second meiotic metaphase (MII) oocytes were obtained (17). Since the metabolic dynamics and required nutrients are not entirely the same in different stages of follicular development, optimization of each step is needed to achieve a higher maturation rate and better oocyte quality, based on the sequential culture system. In this paper, the effects of various additives on different stages are demonstrated, and the current research progress of different stages of human follicular IVC are reviewed, respectively.

## Initial Stage: *In vitro* Activation (IVA) of Primordial Follicles

Primordial follicle activation is the key initial step of oocyte development, involving the interaction of inhibitory factors, stimulating factors, and maintenance factors (18). Early studies showed that the viability of primordial follicles isolated from thawed human ovarian cortical tissues was comparable with that from fresh tissue. However, a lower survival rate was observed in IVC of isolated primordial follicles (19, 20). Consequently, a consensus has been reached that the culture system based on primordial follicles needs to start from human ovarian cortex tissue, not isolated primordial follicles (17, 20–23).

Several pathways, including the phosphatidylinositol 3 kinase (PI3K) pathway, the mechanistic target of rapamycin complex 1 (mTORC1) pathway, and the p27Kip1 (p27)-cyclin dependent kinase (CDK) system, have been shown to regulate the activation of dormant oocytes in the mammalian ovary (18). Some components in these pathways were well-studied and used as regulatory targets for IVC optimization.

The PI3K-PTEN-AKT-FOXO3 signaling pathway is the main non-gonadotrophic growth factor signaling pathway regulating the growth and differentiation of ovarian follicles (24–27). The phosphatase and tensin homolog deleted on chromosome 10 (PTEN), as a negative regulator of PI3K, inhibits the levels of phosphatidylinositol 3,4,5-triphosphate (PIP3), leading to suppressed activation of PI3K signaling (28, 29). The Forkhead box O3 (FOXO3), a downstream effector, also regulates this pathway negatively and suppresses follicle activation, based on mice experiments (24, 30). In women with premature ovarian insufficiency (POI) or premature ovarian failure (POF), mutation in FOXO3 genes was identified and lower FOXO3a expression in ovarian tissue was detected (31, 32). Another component is the mTORC1, a regulator of cell growth and proliferation, involved in promoting primordial follicle activation (33). In previous studies, IVA of primordial follicles was promoted through regulating the pathway by mTOR and PI3K activator, thus improving ART outcomes of patients with POI (26, 34). PTEN inhibition of human ovarian tissue before auto-transplantation has been proved to significantly enhance activation of residual follicles in POI patients, and healthy baby was born (35). Noteworthily, more than 50% POI patients in this study contained no residual follicle and did not respond to the IVA agents, limiting the application of IVA procedure in infertility treatment.

During IVC of the human ovarian cortex, more primordial follicles were activated, and more secondary follicles were isolated with PTEN inhibition. Unfortunately, the survival and development of isolated follicles were poor (26, 36), which may be related to the effect of PTEN inhibition on the DNA repair mechanism in follicles (12). The study involving IVC of bovine ovarian cortex demonstrated that PTEN inhibition activated bovine non-growing follicles, but simultaneously increased DNA damage and reduced DNA repair response (27). Recently, a drug-free IVA method was used in infertility treatment for POI patient, leading to successful pregnancies (37). This method may eliminate the negative effect of activation agents on follicles, but it may simultaneously reduce the yield of follicles, which makes it insufficient for subsequent culture use.

The activation rate of primordial follicles varies with different culture conditions, which seems to be related to the preparation of the ovarian cortical tissue, indicating that mechanical signal was also an important influencing factor (10). The Hippo signaling pathway, which is known for its role in conserving optimal organ size *via* growth inhibition, consists of several negative growth regulators acting in a kinase cascade that ultimately phosphorylates and inactivates key Hippo signaling effectors, Yes-associated protein (YAP)/transcriptional coactivator with PDZ-binding motif (TAZ) (26, 38). Fragmentation of human ovarian cortical tissue can dramatically promote the activation of primordial follicles, which was proved to be the result of interruption of Hippo signal pathway. Ovarian fragmentation increased actin polymerization and disrupted Hippo signaling by decreasing pYAP levels together with increased nuclear localization of YAP, leading to increased expression of CCN growth factors and BIRC apoptosis inhibitors (26, 35). Intuitively, follicles at the most advanced developmental stage were observed at the periphery of the tissue, close to the section site (26). Taken together, the activation of follicles can be significantly promoted by intervening the important components of PI3K pathway, while the Hippo pathway is blocked through the fragmentation of the ovarian cortex (9, 25–27).

At present, common basic media for IVA include MEM-alpha, Waymouth's medium, D-MEM, and McCoys 5a (17, 34, 39, 40). Different laboratories seem to choose the medium used according to their preferences and practices, because no study comparing all media exists. In an early research, two complex media (α-MEM and Waymouth's) were compared with a simple salt solution (EBSS), all of which contained 10% human serum. Results showed that in ovarian tissue cultured in α-MEM, follicle growth was significantly greater and development was improved compared to that seen in follicles cultured with Waymouth's medium. A proportion of follicles cultured with both α-MEM and Waymouth's media reached secondary stages on day 10 of culture. However, only follicles cultured with α-MEM reached preantral stages until days 15 (41). Both media contained amino acids, vitamins, and inorganic salts, but α-MEM has some unique components, including alanine, serine, lipoic acid, ribonucleosides, and deoxyribonucleosides. Meanwhile, the vitamin concentration in α-MEM are higher than that in Waymouth's medium. Unfortunately, the study did not discuss the specific reason of different effects of the two media. It would be interesting to investigate how various nutrients in different media act on primordial follicle IVA.

Further understanding of effect of specific factors on the activation process was another hot topic to improve the quantity and quality of IVC follicles. For example, on the basis of α-MEM, the culture system with human albumin and ITS (insulin, transferrin, selenium) is more favorable to the activation and development of human follicles, leaving less atretic follicles, than that with serum alone. And the addition of follicle stimulating hormone (FSH) to the media greatly reduces atresia and increases follicle diameter simultaneously (41). However, the findings in different reports could be contradictory. One study in 2005 showed that human follicular recruitment and activation were promoted in culture media with 300 ng/ml anti-Mullerian hormone (AMH) (40), while another research in 2006 disclosed the inhibitory effect of 100 ng/ml AMH on human follicles activation (42). The reason for the difference between findings was unclear. It is possible that AMH influences primordial follicle activation in a dose-dependent manner as shown in a study using an ovine pre-pubertal ovarian cortex culture system (43). In fact, no unified standard for the timing and dosage of important additives has been developed. Currently, a consensus has been reached that the interaction between oocytes and somatic cells is essential for the activation and development of follicles (18). The intercellular communication is influenced by the members of TGF beta superfamily, especially the oocyte specific factors: growth differentiation factor (GDF)-9 and bone morphogenetic protein (BMP)-15 (44). In human follicles, GDF-9 and BMP-15 are expressed both in oocytes and cumulus granulosa cells. The increase of mRNA levels in cumulus cells is related to the oocyte maturity and fertilization rate (45), and the addition of GDF-9 or BMP-15 in IVC system can promote the activation of human primordial follicles, with seemingly more beneficial effects of GDF9 (46). Other factors, such as basic fibroblast growth factor (bFGF), kit ligand (KITL), keratinocyte growth factor (KGF), leukemia inhibitory factor (LIF), stem cell factor (SCF), vascular endothelial growth factor (VEGF), and cyclic adenosine monophosphate (cAMP), have also been proved to improve the activation and survival of cultured follicles *in vitro* (47–49). Shown in Table 1.

**Table 1:** Reported additives in human follicle *in vitro* activation stage.

**Additives**	**Species**	**Dose**	**Mechanism**	**Effect**	**Detection method**
740Y-P (26, 34, 35)	Human	150 μg/ml	PI3K/AKT activator	More follicles activated, no survival difference; Increase in p-PTEN levels and Akt and rpS6 phosphorylation; Higher serum AMH; Healthy baby achieved.	Histological analysis; TUNEL apoptosis assay; Immunohistochemistry for Ki67, GDF9, rtPCR for TSC1, LATS1, Kit Ligand; E2 and AMH assay.
bpV(HOpic) (26, 34, 35)	Human	30 μM	PTEN inhibitor		
bpV (HOpic) (36)	Human	1 μM	PTEN inhibitor	More follicles activated, with limited growth and reduced survival of isolated follicles.	Histological analysis; Immunohistochemistry for FOXO3.
rhAMH (40)	Human	300 ng/ml	May rescue some follicles from entering atresia	Enhance follicles recruitment, survival and growth.	Histological analysis
rrAMH (42)	Human	100 ng/ml	Act as a negative paracrine feedback signal	Suppress the initiation of the growth of primordial follicles, without detrimental effect on viability or follicle density.	Histological analysis
FSH (41)	Human	300 mIU/ml	Prevent apoptotic atresia; Mitogenic function	Reduce the proportion of atretic follicles; Increase follicle size and healthy follicles.	Histological analysis
HSA+ITS (41)	Human	2.5% HSA; 1%ITS*	Promote cell proliferation; Act as free-radical scavengers.	Reduce the proportion of atretic follicles; Increase follicle size and healthy follicles.	Histological analysis
GDF9 (46)	Human	10 ng/ml	BMP15 activates the intracellular signal-mediated pathways Smad1, Smad5, and Smad8; GDF9 activates Smad2 and Smad3	More follicles activated;	Histological analysis; Immunohistochemistry
		100 ng/ml		More follicles activated; increase PCNA expression;	
BMP15 (46)	Human	10 ng/ml		More follicles activated;	
		100ng/ml		Increase PCNA expression;	
cAMP (48)	Human	0.5 mM	Induce formation of the FSH receptor, as an intracellular second messenger	More follicles activated;	Histological analysis

* *1% ITS includes 10 mg/ml insulin, 5.5 mg/ml transferrin, and 6.7 ng/ml sodium selenite*.

*rhAMH, recombinant human anti-Mullerian hormone; rrAMH, recombinant rat anti-Mullerian hormone; FSH, follicle stimulating hormone; HSA, human serum albumin; GDF9, growth differentiation factor 9; BMP15, bone morphogenetic protein 15; cAMP, cyclic adenosine monophosphate*.

## Development Stage: *In vitro* Growth (IVG) of Separated Secondary Follicles

As mentioned above, the culture system based on primordial follicles needs to start from human ovarian cortex tissue, in which the activated follicles are capable of developing to secondary stage, with multi-layer granular cells. However, further development will be inhibited if secondary follicles are not separated from the cortex (17, 23, 39). Secondary follicles can be separated by an enzymatic method, mechanical method or combination of the two methods. Mechanical isolation, with a fine syringe needle, has been commonly used for follicle isolation (17, 50, 51). This method preserves the natural follicular morphology, maintaining an intact theca cell layer and a normal basement membrane. However, it is inevitably laborious and inefficient (52), especially for denser human ovarian tissue. Enzymatic follicle isolation typically involves collagenase and DNase enzymolysis, by which the COCs were obtained from mice ovaries, and offsprings were produced following appropriate culture, indicating that intact follicle structure is not an absolute requirement during follicle IVC (53). A later study on molecular level showed that theca cells of mechanically-isolated secondary follicles induced the expression of IGF1 and promoted follicular growth in mice, while many follicles with theca cells still showed no growth during culture (54). It seems that theca cells affect follicular development to some extent, but may not be the only and necessary factor, as long as migration of granulosa cells from oocyte could be minimized (53). Interestingly, one study even demonstrated that although incubated with enzymes for 1 h, most enzymatically isolated follicles contained more theca cells compared with mechanically isolated follicles, which is contrary to our general thought (55). However, the influence of the operating technology of the experimenters cannot be excluded. Most recently, a research compared four isolation methods (two mechanical and two enzymatic) with mice, concluding that mechanical methods were preferable, with better follicular growth, survival, and MII rate. In addition, mincing isolation with a cell dissociation kit (SigmaAldrich) was more effective than conventional mechanical isolation (56). Overall, there is no one method that is absolutely superior to the others. And considering the difference between ovaries of mice and humans, both methods need to be improved and well-designed comparative researches on human samples are required.

IVG of isolated secondary follicles also depends on oocyte somatic interaction (57). At this stage, addition of activin and low-dose FSH, based on IVA medium, can help to maintain the intercellular connection, improve the quality of oocytes and promote the formation of antrum, which has been verified in dog, bovine, and human experiments (17, 39, 58, 59). Addition of 200 ng/ml bFGF in IVG medium also promotes the development of human ovarian early follicles (60), as shown in Table 2.

**Table 2:** Reported additives in human follicle *in vitro* growth stage.

**Additives**	**Species**	**Dose**	**Mechanism**	**Effect**	**Detection method**
Actin A (39)	Human	100 ng/ml	Regulate granulosa cell growth and differentiation	Increase follicles diameter; Higher estradiol secretion; More healthy follicles	Histological analysis; Diameter measurements; Detection of estradiol in culture media by enzyme immunoassay
FSH (59)	Human	1.5 U/ml	Increase E2 production	Promote follicle antrum formation and growth	Histological analysis; E2 assay.
bFGF (60)	Human	200 ng/ml	Promote GC proliferation, suppress apoptosis in preantral follicles, and enhance early follicle cell differentiation.	Higher diameter and survival rate, with good viability.	Histological analysis; Confocal analysis.

*FSH, follicle stimulating hormone; bFGF, basic fibroblast growth factor*.

In addition to optimizing the composition of culture medium, the physical environment of isolated follicle culture is also widely concerned. Conventional planar 2D culture plates may destroy the connection between human oocytes and granulosa cells, and inhibit the development of follicles (61). Therefore, some 3D culture systems were established to support the IVG of pre-antral follicles, and tissue engineering technology was also applied to this stage (62). Human preantral follicles can be encapsulated in bio-matrices such as alginate to complete growth and development *in vitro* (63). Alginate is a polysaccharide found in algal cell walls, which can form rigid and controllable gel. Studies have shown poor growth of mouse follicles embedded in 1% alginate gels (64), while 0.5% alginate gel supports the growth of human oocytes well (63). The research using microfluidic technology to encapsulate follicles also proved the function of mechanical heterogeneity in follicle development and ovulation (65). This indicated the effect of rigidity of the gel and the significance of balancing flexibility of the gel to accommodate cell proliferation with its rigidity to avoid collapse of the follicle structure (66). Other scaffolds such as de-cellularised ovarian tissue and 3D microporous scaffolds were also applied to support human pre-antral follicle growth (67, 68).

In previous multi-step IVC system of human follicles, isolated secondary follicles were directly placed in a 96 well V-bottomed plate, without any extracellular matrix or scaffold. Sixty-two percent of the follicles completed the process of growth, differentiation and follicle antrum formation, but only 8% of the follicles finally reached MII stage (17). Another team's research showed that the follicles encapsulated in alginate can reach 110 μm in diameter after 30 days of culture, while the oocytes in these follicles cannot reach MII stage (63). Considering that human immature follicles gradually move from the rigid collagen-dense cortex zone to the less dense perimedullary region as they grow (69, 70), this team released a portion of follicles from the alginate hydrogels at antral stage, and then continued the culture in low-attachment plates for up to 40 days in further study. Results showed that follicles cultured with only alginate encapsulation produced oocytes that either remained in the germinal vesicle (GV) stage or degenerated, while 20% (four out of 20) of follicles cultured using the two-step strategy produced meiotic competent MII oocytes (61). In the future, more research is needed to compare different culture environments to find out which one best supports the development of follicles *in vitro*.

## Maturation Stage: *In vitro* Maturation (IVM) of Oocytes

Since human immature oocytes were cultured by IVM technology, resulting in a live birth after fertilization in 1991 (71), many studies regarding the establishment and optimization of the IVM system have been reported. The source of oocytes selected for IVM seems to be a crucial factor as the successful maturation of immature oocytes is lower than that of oocytes obtained from stimulated ovaries (72, 73). The developmental competence of IVG derived oocytes is different from that of natural or controlled ovulation cycle derived oocytes. Therefore, if IVG is to be used in clinical practice, IVM conditions need to be optimized (74).

In 2008, Telfer et al. developed a two-step culture system, which initially demonstrated that individual human pre-antral follicles grown *in vitro* from the primordial stage, within human ovarian cortical strips, can be isolated and have the potential to grow to the antral stage (39). From then, scientists have been working on bridging the gap between IVG of follicles and IVM of oocytes. The oocytes produced by IVG need to be transferred to the maturation medium when they reach a certain stage, so as to complete the final meiotic resumption and maturation. In 2015, a study comparing alginate encapsulation culture and two-step culture of isolated follicles revealed that the diameters of oocytes that reached the MII stage was not significantly different from that of oocytes that remained in GV stage, indicating that the oocyte meiotic competence could be achieved although the terminal follicle size cannot reach the size of preovulatory follicles *in vivo* (61). In the later modified multi-step IVC system, follicular antrum formation was observed within 6–8 days of isolated follicle culture, and at the time COCs were removed for subsequent culture, without waiting for the follicle diameter to increase to the preovulatory follicle level. After IVM, oocytes with a diameter over 100 μm were obtained and few of them developed to MII stage (17). These results indicated that a more direct and efficient IVC process could be achieved based on the development of oocytes rather than the diameter of follicles.

However, the maturation rates of IVG derived oocytes were still not ideal. In the most recent multi-step culture system, 32 COC from IVG were selected for IVM, and nine oocytes emitted polar bodies after 24 h of maturation (17). Optimization is required not only for the IVM stage, but also for the whole culturing course. Nevertheless, most of the studies focused merely on the maturation stage of the oocytes, so the oocytes used in the experiments were obtained mainly from immature oocytes in the COH cycle, while IVG derived oocyte maturation was less reported. While almost all the literature on IVM optimization was based on oocytes harvested from stimulated ovaries, a review of the results of these literature still helps to optimize IVM following IVG.

Common basic media for IVM include α-MEM, SAGE, TCM-199 (17, 61, 75), although which medium is better for oocyte maturation is still uncertain. To explore the best conditions of IVM, a good understanding of the mechanism of oocyte maturation is needed. cAMP plays a crucial role in regulating oocyte maturation. The granulosa cells in the outer layer contain C-type natriuretic peptide (CNP), while the natriuretic peptide receptor (NPR2) is expressed in the cumulus cells around the oocytes. The paracrine factors of oocytes promote the activation of NPR2 in the cumulus cells, and the CNP binds to the NPR2 receptor in the cumulus cells to produce cyclic guanosine monophosphate (cGMP), which enters the oocytes through gap junctions, inhibits the activity of phosphodiesterase (PDE3A), maintains a high level of cAMP, and thus keeps the meiosis arrest of oocytes. When PDE3A is activated by LH, the cAMP level in oocytes would be down-regulated, resulting in resumption from meiotic arrest of the immature oocytes in the GV stage or the first meiotic metaphase. Subsequently, the oocytes reach MII stage and complete maturation (76).

Accordingly, synchronization of nuclear and cytoplasmic maturation processes within the oocyte could be achieved through controlling COC cAMP level. Experiments showed that artificial regulation of meiotic resumption by cAMP elevating agents improved subsequent human oocyte developmental competence (77, 78). Combined treatment of cilostamide (PDE3 specific inhibitor) and forskolin (adenylate cyclase activator) positively influences the IVM oocyte developmental competence (79). Using CNP in the medium increased the meiotic maturation rate of IVM human oocytes (80). Some other factors also have been proved to promote oocyte maturation or benefit subsequent processes (Table 3). The addition of GDF9 had no effect on maturation rate, while it significantly enhanced fertilization, embryo formation, and viability rates of blastocysts (82). And the growth hormone (GH) was applied to improve the IVM maturation rate of human oocytes (81). More specifically, melatonin had no significant effect on the maturation rate of oocytes, while it increased the blastocyst formation rate after fertilization from 24.5 to 49.3%, by protecting mitochondrial function (75). High-glucose concentrations altered DNA methylation levels of Peg3 and adiponectin in human IVM oocytes (83).

**Table 3:** Reported additives in human follicle *in vitro* maturation stage.

**Additives**	**Species**	**Dose**	**Mechanism**	**Effect**	**Detection method**
Melatonin (75)	Human	10 μmol/L	Maintain the mitochondrial oxidative phosphorylation function in the oocytes by effectively inhibiting environmental stress, providing sufficient ATP for subsequent embryo development.	Increased high-quality blastocyst formation rate, with low aneuploidy rate.	Blastocyst grading and examination of aneuploidy; Single-cell RNA-sequence analysis; Detection of ROS and calcium levels in human oocytes; Mitochondrial function assay in human oocytes
GH (81)	Human	200 ng/ml	Accelerate meiotic progression, balance redox homeostasis of cellular environment, and promote oocyte developmental competence	Promote maturation of human oocytes	Oocyte RNA Sequencing; Real-Time PCR for Validation
CNP (80)	Human	25 nM	Binds the Natriuretic peptide receptor 2 (NPR2), induces the production of cGMP	Increase the meiotic maturation rate	Evaluation of blastocyst yield; Documentation of aneuploidy rate in blastocysts; Assessment of cumulus-oocyte TZPs; Evaluation of chromatin configuration in GV oocytes
GDF9 (82)	Human	200 ng/mL	Promote cumulus expansion	Increase the embryo formation rate and blastocyst viability	Assessment of maturation, fertilization, embryo formation, blastocyst formation rate, and blastocyst viability (by Propidium iodide/Hoechst staining).
Cilostamide (79)	Human	20 μM	PDE3 specific inhibitor	Positively influence oocyte developmental competence by exhibiting a synergistic effect on the prevention of gap junction communication loss and resumption of meiosis	Intercellular gap junction communication and maturational status were examined by fluorophotometry;
Forskolin (79)	Human	50 μM	Adenylate cyclase activator		

*GH, growth hormone; CNP, C-type natriuretic peptide*.

## Conclusion and Prospects

Based on the animal experiments and the basic human follicle IVC system established, the concept of achieving MII oocytes from human primordial follicles through IVC to obtain offspring after *in vitro* fertilization (IVF) seems promising. However, currently, the clinical use of human follicular IVC systems is still limited by low MII rates, ambiguous fertilization capacities, and unknown safety. Based on the information provided by this review, future studies may focus on the following: (1) IVA: clarifying the safe concentration and safe exposure duration of existing activators, or further exploring the drug-free activation; (2) IVG: exploring the optimal method and timing of human follicle isolation, and developing the 3-dimension system, combined with bioengineering technology, such as microfluidic technology (84, 85), to simulate the *in vivo* follicular development environment; (3) IVM: establishing a whole-course culture system to improve the maturation rate and developmental potential of IVG-derived oocytes, in addition to studying this stage alone; (4) to explore the fertilization ability and embryo developmental potential of mature oocytes obtained by IVC within ethical agreements.

In conclusion, IVC is likely to provide an ideal option for the fertility preservation of young cancer patients and for the infertility treatment of patients with POI or POF in the future. However, before this technology is applied to clinical practice, a large number of studies are required to optimize each stage of follicular development *in vitro*, establishing a relatively unified, standard, efficient and safe culture system.

## Author Contributions

All authors have contributed to the preparation of this manuscript, read, and approved the manuscript.

## Conflict of Interest

The authors declare that the research was conducted in the absence of any commercial or financial relationships that could be construed as a potential conflict of interest.

## References

[1] Siegel RL Miller KD Jemal A Cancer statistics, 2020. CA Cancer J Clin. (2020) 70:7–30. 10.3322/caac.21590 31912902

[2] Anderson RA Baird DT. The development of ovarian tissue cryopreservation in Edinburgh: translation from a rodent model through validation in a large mammal and then into clinical practice. Acta Obstet Gynecol Scand. (2019) 98:545–9. 10.1111/aogs.13560 30723908

[3] Anderson RA Wallace WHB Telfer EE. Ovarian tissue cryopreservation for fertility preservation: clinical and research perspectives. Hum Reprod Open. (2017) 2017:hox001. 10.1093/hropen/hox001 30895221 PMC6276668

[4] Dolmans MM Luyckx V Donnez J Andersen CY Greve T. Risk of transplanting malignant cells with transplanted frozen-thawed ovarian tissue. Fertil Steril. (2013) 99:1514–22. 10.1016/j.fertnstert.2013.03.027 23541406

[5] de Vos M Smitz J Woodruff TK. Fertility preservation in women with cancer. Lancet. (2014) 384:1302–10. 10.1016/S0140-6736(14)60834-5 25283571 PMC4270060

[6] Telfer EE Zelinski MB. Ovarian follicle culture: advances and challenges for human and nonhuman primates. Fertil Steril. (2013) 99:1523–33. 10.1016/j.fertnstert.2013.03.043 23635350 PMC3929501

[7] Telfer EE. Future developments: *in vitro* growth (IVG) of human ovarian follicles. Acta Obstet Gynecol Scand. (2019) 98:653–8. 10.1111/aogs.13592 30801653

[8] Anderson RA Mitchell RT Kelsey TW Spears N Telfer EE Wallace WH. Cancer treatment and gonadal function: experimental and established strategies for fertility preservation in children and young adults. Lancet Diabetes Endocrinol. (2015) 3:556–67. 10.1016/S2213-8587(15)00039-X 25873571

[9] Hsueh AJ Kawamura K Cheng Y Fauser BC. Intraovarian control of early folliculogenesis. Endocr Rev. (2015) 36:1–24. 10.1210/er.2014-1020 25202833 PMC4309737

[10] Shah JS Sabouni R Cayton Vaught KC Owen CM Albertini DF Segars JH. Biomechanics and mechanical signaling in the ovary: a systematic review. J Assist Reprod Genet. (2018) 35:1135–48. 10.1007/s10815-018-1180-y 29691711 PMC6063820

[11] Anderson RA Telfer EE. Being a good egg in the 21st century. Br Med Bull. (2018) 127:83–9. 10.1093/bmb/ldy023 30084904 PMC6127894

[12] Martin JH Aitken RJ Bromfield EG Nixon B. DNA damage and repair in the female germline: contributions to ART. Hum Reprod Update. (2019) 25:180–201. 10.1093/humupd/dmy040 30541031

[13] Eppig JJ O'Brien MJ. Development *in vitro* of mouse oocytes from primordial follicles. Biol Reprod. (1996) 54:197–207. 10.1095/biolreprod54.1.197 8838017

[14] O'Brien MJ Pendola JK Eppig JJ. A revised protocol for *in vitro* development of mouse oocytes from primordial follicles dramatically improves their developmental competence. Biol Reprod. (2003) 68:1682–6. 10.1095/biolreprod.102.013029 12606400

[15] Hirao Y Naruse K Kaneda M Somfai T Iga K Shimizu M. Production of fertile offspring from oocytes grown *in vitro* by nuclear transfer in cattle. Biol Reprod. (2013) 89:57. 10.1095/biolreprod.113.109439 23884646

[16] Herta AC Lolicato F Smitz JEJ. *In vitro* follicle culture in the context of IVF. Reproduction. (2018) 156:F59–73. 10.1530/REP-18-0173 29980584

[17] McLaughlin M Albertini DF Wallace WHB Anderson RA Telfer EE. Metaphase II oocytes from human unilaminar follicles grown in a multi-step culture system. Mol Hum Reprod. (2018) 24:135–42. 10.1093/molehr/gay002 29390119

[18] Zhang H Liu K. Cellular and molecular regulation of the activation of mammalian primordial follicles: somatic cells initiate follicle activation in adulthood. Hum Reprod Update. (2015) 21:779–86. 10.1093/humupd/dmv037 26231759

[19] Oktay K Nugent D Newton H Salha O Chatterjee P Gosden RG. Isolation and characterization of primordial follicles from fresh and cryopreserved human ovarian tissue. Fertil Steril. (1997) 67:481–6. 10.1016/S0015-0282(97)80073-8 9091334

[20] Hovatta O Silye R Abir R Krausz T Winston RM. Extracellular matrix improves survival of both stored and fresh human primordial and primary ovarian follicles in long-term culture. Hum Reprod. (1997) 12:1032–6. 10.1093/humrep/12.5.1032 9194661

[21] Picton HM Gosden RG. *In vitro* growth of human primordial follicles from frozen-banked ovarian tissue. Mol Cell Endocrinol. (2000) 166:27–35. 10.1016/S0303-7207(00)00294-X 10989205

[22] Gosden RG Mullan J Picton HM Yin H Tan SL. Current perspective on primordial follicle cryopreservation and culture for reproductive medicine. Hum Reprod Update. (2002) 8:105–10. 10.1093/humupd/8.2.105 12099625

[23] Anderson RA McLaughlin M Wallace WH Albertini DF Telfer EE. The immature human ovary shows loss of abnormal follicles and increasing follicle developmental competence through childhood and adolescence. Hum Reprod. (2014) 29:97–106. 10.1093/humrep/det388 24135076 PMC3860895

[24] Reddy P Liu L Adhikari D Jagarlamudi K Rajareddy S Shen Y. Oocyte-specific deletion of Pten causes premature activation of the primordial follicle pool. Science. (2008) 319:611–3. 10.1126/science.1152257 18239123

[25] Li J Kawamura K Cheng Y Liu S Klein C Liu S. Activation of dormant ovarian follicles to generate mature eggs. Proc Natl Acad Sci USA. (2010) 107:10280–4. 10.1073/pnas.1001198107 20479243 PMC2890455

[26] Grosbois J Demeestere I. Dynamics of PI3K and hippo signaling pathways during *in vitro* human follicle activation. Hum Reprod. (2018) 33:1705–14. 10.1093/humrep/dey250 30032281

[27] Maidarti M Clarkson YL McLaughlin M Anderson RA Telfer EE. Inhibition of PTEN activates bovine non-growing follicles *in vitro* but increases DNA damage and reduces DNA repair response. Hum Reprod. (2019) 34:297–307. 10.1093/humrep/dey354 30521029 PMC6343469

[28] Salmena L Carracedo A Pandolfi PP. Tenets of PTEN tumor suppression. Cell. (2008) 133:403–14. 10.1016/j.cell.2008.04.013 18455982

[29] Carolyn A Worby Jack E Dixon PTEN. Annu Rev Biochem. (2014) 83:641–69. 10.1146/annurev-biochem-082411-113907 24905788

[30] Castrillon DH Miao L Kollipara R Horner JW DePinho RA. Suppression of ovarian follicle activation in mice by the transcription factor Foxo3a. Science. (2003) 301:215–8. 10.1126/science.1086336 12855809

[31] Thanatsis N Kaponis A Koika V Georgopoulos NA Decavalas GO Reduced Foxo3a, FoxL2, and p27 mRNA expression in human ovarian tissue in premature ovarian insufficiency. Hormones. (2019) 18:409–15. 10.1007/s42000-019-00134-4 31637660

[32] Wendy J Watkins Alexandra J Umbers Kathryn J Woad Sarah E Harris Ingrid M Winship Ksenija Gersak. Mutational screening of FOXO3A and FOXO1A in women with premature ovarian failure. Fertil Steril. (2006) 86:1518–21. 10.1016/j.fertnstert.2006.03.054 16979636

[33] Adhikari D Liu K mTOR signaling in the control of activation of primordial follicles. Cell Cycle. (2010) 9:1673–74. 10.4161/cc.9.9.11626 20404510

[34] Suzuki N Yoshioka N Takae S Sugishita Y Tamura M Hashimoto S. Successful fertility preservation following ovarian tissue vitrification in patients with primary ovarian insufficiency. Hum Reprod. (2015) 30:608–15. 10.1093/humrep/deu353 25567618

[35] Kawamura K Cheng Y Suzuki N Deguchi M Sato Y Takae S. Hippo signaling disruption and Akt stimulation of ovarian follicles for infertility treatment. Proc Natl Acad Sci USA. (2013) 110:17474–9. 10.1073/pnas.1312830110 24082083 PMC3808580

[36] McLaughlin M Kinnell HL Anderson RA Telfer EE. Inhibition of phosphatase and tensin homologue (PTEN) in human ovary *in vitro* results in increased activation of primordial follicles but compromises development of growing follicles. Mol Hum Reprod. (2014) 20:736–44. 10.1093/molehr/gau037 24830779 PMC4106636

[37] Ferreri J Fàbregues F Calafell JM Solernou R Borrás A Saco A. Drug-free *In-vitro* activation of follicles and fresh tissue autotransplantation as a therapeutic option in patients with primary ovarian insufficiency. Reprod Biomed Online. (2020) 40:254–60. 10.1016/j.rbmo.2019.11.009 31956062

[38] Badouel C McNeill H. SnapShot: the hippo signaling pathway. Cell. (2011) 145:484.e1. 10.1016/j.cell.2011.04.009 21529719

[39] Telfer EE McLaughlin M Ding C Thong KJ. A two-step serum-free culture system supports development of human oocytes from primordial follicles in the presence of activin. Hum Reprod. (2008) 23:1151–8. 10.1093/humrep/den070 18326514

[40] Schmidt KL Kryger-Baggesen N Byskov AG Andersen CY. Anti-Mullerian hormone initiates growth of human primordial follicles *in vitro*. Mol Cell Endocrinol. (2005) 234:87–93. 10.1016/j.mce.2004.12.010 15836957

[41] Wright CS Hovatta O Margara R Trew G Winston RM Franks S. Effects of follicle-stimulating hormone and serum substitution on the *In-vitro* growth of human ovarian follicles. Hum Reprod. (1999) 14:1555–62. 10.1093/humrep/14.6.1555 10357975

[42] Carlsson IB Scott JE Visser JA Ritvos O Themmen AP Hovatta O. Anti-Müllerian hormone inhibits initiation of growth of human primordial ovarian follicles *in vitro*. Hum Reprod. (2006) 21:2223–7. 10.1093/humrep/del165 16720622

[43] Bertoldo MJ Bernard J Duffard N Tsikis G Alves S Calais L. Inhibitors of c-Jun phosphorylation impede ovine primordial follicle activation. Mol. Hum. Reprod. (2016) 22:338–49. 10.1093/molehr/gaw012 26908644

[44] Sanfins A Rodrigues P Albertini DF. GDF-9 and BMP-15 direct the follicle symphony. J Assist Reprod Genet. (2018) 35:1741–50. 10.1007/s10815-018-1268-4 30039232 PMC6150895

[45] Li Y Li RQ Ou SB Zhang NF Ren L Wei LN. Increased GDF9 and BMP15 mRNA levels in cumulus granulosa cells correlate with oocyte maturation, fertilization, and embryo quality in humans. Reprod Biol Endocrinol. (2014) 12:81. 10.1186/1477-7827-12-81 25139161 PMC4153897

[46] Kedem A Fisch B Garor R Ben-Zaken A Gizunterman T Felz C. Growth differentiating factor 9(GDF9) and bone morphogenetic protein 15 both activate development of human primordial follicles *in vitro*, with seemingly more beneficial effects of GDF9. J Clin Endocrinol Metab. (2011) 96:E1246–54. 10.1210/jc.2011-0410 21632818

[47] Bertoldo MJ Walters KA Ledger WL Gilchrist RB Mermillod P Locatelli Y. *In-vitro* regulation of primordial follicle activation: challenges for fertility preservation strategies. Reprod Biomed Online. (2018) 36:491–9. 10.1016/j.rbmo.2018.01.014 29503209

[48] Zhang P Louhio H Tuuri T Sjöberg J Hreinsson J Telfer EE. *In vitro* effect of cyclic adenosine 3', 5'-monophosphate (cAMP) on early human ovarian follicles. J Assist Reprod Genet. (2004) 21:301–6. 10.1023/B:JARG.0000043704.10845.87 15568331 PMC3455439

[49] Asadi E Najafi A Moeini A Pirjani R Hassanzadeh G Mikaeili S. Ovarian tissue culture in presence of VEGF and fetuin stimulates follicle growth and steroidogenesis. J Endocrinol. (2017) 232:205–19. 10.1530/JOE-16-0368 27852727

[50] Cortvrindt R Smitz J van Steirteghem AC. *In-vitro* maturation, fertilization and embryo development of immature oocytes from early preantral follicles from prepuberal mice in a simplified culture system. Hum Reprod. (1996) 11:2656–66. 10.1093/oxfordjournals.humrep.a019188 9021369

[51] Spears N Boland NI Murray AA Gosden RG. Mouse oocytes derived from *in vitro* grown primary ovarian follicles are fertile. Hum Reprod. (1994) 9:527–32. 10.1093/oxfordjournals.humrep.a138539 8006146

[52] Telfer EE Binnie JP McCaffery FH Campbell BK. *In vitro* development of oocytes from porcine and bovine primary follicles. Mol Cell Endocrinol. (2000) 163:117–23. 10.1016/S0303-7207(00)00216-1 10963883

[53] Eppig John J Schroeder Allen C. Capacity of mouse oocytes from preantral follicles to undergo embryogenesis and development to live young after growth, maturation, and fertilization *in vitro*. Biol Reprod. (1989) 41:268–76. 10.1095/biolreprod41.2.268 2508774

[54] Shiomi-Sugaya N Komatsu K Wang J Yamashita M Kikkawa F Iwase A. Regulation of secondary follicle growth by theca cells and insulin-like growth factor 1. J Reprod Dev. (2015) 61:161–8. 10.1262/jrd.2014-107 25740252 PMC4498370

[55] Demeestere I Delbaere A Gervy C Van Den Bergh M Devreker F Englert Y. Effect of preantral follicle isolation technique on *in vitro* follicular growth, oocyte maturation and embryo development in mice. Hum Reprod. (2002) 17:2152–59. 10.1093/humrep/17.8.2152 12151451

[56] Kim EJ Lee J Youm HW Kim SK Lee JR Suh CS. Comparison of follicle isolation methods for mouse ovarian follicle culture *in vitro*. Reprod Sci. (2018) 25:1270–8. 10.1177/1933719117737851 29113581

[57] Li R Albertini DF. The road to maturation: somatic cell interaction and self-organization of the mammalian oocyte. Nat Rev Mol Cell Biol. (2013) 14:141–52. 10.1038/nrm3531 23429793

[58] McLaughlin M Bromfield JJ Albertini DF Telfer EE. Activin promotes follicular integrity and oogenesis in cultured pre-antral bovine follicles. Mol Hum Reprod. (2010) 16:644–53. 10.1093/molehr/gaq021 20203128 PMC2930516

[59] Abir R Franks S Mobberley MA Moore PA Margara RA Winston RM. Mechanical isolation and *in vitro* growth of preantral and small antral human follicles. Fertil Steril. (1997) 68:682–8. 10.1016/S0015-0282(97)00264-1 9341611

[60] Wang TR Yan LY Yan J Lu CL Xia X Yin TL. Basic fibroblast growth factor promotes the development of human ovarian early follicles during growth *in vitro*. Hum Reprod. (2014) 29:568–76. 10.1093/humrep/det465 24408318

[61] Xiao S Zhang J Romero MM Smith KN Shea LD Woodruff TK. *In vitro* follicle growth supports human oocyte meiotic maturation. Sci Rep. (2015) 5:17323. 10.1038/srep17323 26612176 PMC4661442

[62] Shea LD Woodruff TK Shikanov A. Bioengineering the ovarian follicle microenvironment. Annu Rev Biomed Eng. (2014) 16:29–52. 10.1146/annurev-bioeng-071813-105131 24849592 PMC4231138

[63] Xu M Barrett SL West-Farrell E Kondapalli LA Kiesewetter SE Shea LD. *In vitro* grown human ovarian follicles from cancer patients support oocyte growth. Hum Reprod. (2009) 24:2531–40. 10.1093/humrep/dep228 19597190 PMC2743446

[64] Heise M Koepsel R Russell AJ McGee EA. Calcium alginate microencapsulation of ovarian follicles impacts FSH delivery and follicle morphology. Reprod Biol Endocrinol. (2005) 3:47. 10.1186/1477-7827-3-47 16162282 PMC1262772

[65] Choi JK Agarwal P Huang H Zhao S He X. The crucial role of mechanical heterogeneity in regulating follicle development and ovulation with engineered ovarian microtissue. Biomaterials. (2014) 35:5122–8. 10.1016/j.biomaterials.2014.03.028 24702961 PMC4016819

[66] Skory RM Xu Y Shea LD Woodruff TK. Engineering the ovarian cycle using *in vitro* follicle culture. Hum Reprod. (2015) 30:1386–95. 10.1093/humrep/dev052 25784584 PMC4447886

[67] Laronda MM Rutz AL Xiao S Whelan KA Duncan FE Roth EW. A bioprosthetic ovary created using 3D printed microporous scaffolds restores ovarian function in sterilized mice. Nat Commun. (2017) 8:15261. 10.1038/ncomms15261 28509899 PMC5440811

[68] Pors SE Ramlose M Nikiforov D Lundsgaard K Cheng J Andersen CY. Initial steps in reconstruction of the human ovary: survival of pre-antral stage follicles in a decellularized human ovarian scaffold. Hum Reprod. (2019) 34:1523–35. 10.1093/humrep/dez077 31286144

[69] Tingen C Kim A Woodruff TK. The primordial pool of follicles and nest breakdown in mammalian ovaries. Mol Hum Reprod. (2009) 15:795–803. 10.1093/molehr/gap073 19710243 PMC2776475

[70] Woodruff TK Shea LD. A new hypothesis regarding ovarian follicle development: ovarian rigidity as a regulator of selection and health. J Assist Reprod Gen. (2011) 28:3–6. 10.1007/s10815-010-9478-4 20872066 PMC3045494

[71] Cha KY Koo JJ Ko JJ Choi DH Han SY Yoon TK. Pregnancy after *in vitro* fertilization of human follicular oocytes collected from nonstimulated cycles, their culture *in vitro* and their transfer in a donor oocyte program. Fertil Steril. (1991) 55:109–13. 10.1016/S0015-0282(16)54068-0 1986950

[72] Nogueira D Sadeu JC Montagut J. *In vitro* oocyte maturation: current status. Semin Reprod Med. (2012) 30:199–213. 10.1055/s-0032-1311522 22585631

[73] Chian RC Uzelac PS Nargund G. *In vitro* maturation of human immature oocytes for fertility preservation. Fertil Steril. (2013) 99:1173–81. 10.1016/j.fertnstert.2013.01.141 23433515

[74] Shirasawa H Terada Y. *In vitro* maturation of human immature oocytes for fertility preservation and research material. Reprod Med Biol. (2017) 16:258–67. 10.1002/rmb2.12042 29259476 PMC5715881

[75] Zou H Chen B Ding D Gao M Chen D Liu Y. Melatonin promotes the development of immature oocytes from the COH cycle into healthy offspring by protecting mitochondrial function. J Pineal Res. (2020) 68:e12621. 10.1111/jpi.12621 31714635

[76] Zhang M Su YQ Sugiura K Xia G Eppig JJ. Granulosa cell ligand NPPC and its receptor NPR2 maintain meiotic arrest in mouse oocytes. Science. (2010) 330:366–9. 10.1126/science.1193573 20947764 PMC3056542

[77] Nogueira D Albano C Adriaenssens T Cortvrindt R Bourgain C Devroey P. Human oocytes reversibly arrested in prophase I by phosphodiesterase type 3 inhibitor *in vitro*. Biol Reprod. (2003) 69:1042–52. 10.1095/biolreprod.103.015982 12773402

[78] Nogueira D Ron-El R Friedler S Schachter M Raziel A Cortvrindt R. Meiotic arrest *in vitro* by phosphodiesterase 3-inhibitor enhances maturation capacity of human oocytes and allows subsequent embryonic development. Biol Reprod. (2006) 74:177–84. 10.1095/biolreprod.105.040485 16207840

[79] Shu YM Zeng HT Ren Z Zhuang GL Liang XY Shen HW. Effects of cilostamide and forskolin on the meiotic resumption and embryonic development of immature human oocytes. Hum Reprod. (2008) 23:504–13. 10.1093/humrep/dem344 18216034

[80] Sánchez F Lolicato F Romero S De Vos M Van Ranst H Verheyen G. An improved IVM method for cumulus-oocyte complexes from small follicles in polycystic ovary syndrome patients enhances oocyte competence and embryo yield. Hum Reprod. (2017) 32:2056–68. 10.1093/humrep/dex262 28938744

[81] Li Y Liu H Yu Q Liu H Huang T Zhao S. Growth hormone promotes *in vitro* maturation of human oocytes. Front Endocrinol. (2019) 10:485. 10.3389/fendo.2019.00485 31396155 PMC6667636

[82] Chatroudi MH Khalili MA Ashourzadeh S Anbari F Shahedi A Safari S. Growth differentiation factor 9 and cumulus cell supplementation in *in vitro* maturation culture media enhances the viability of human blastocysts. Clin Exp Reprod Med. (2019) 46:166–72. 10.5653/cerm.2019.00402 31813208 PMC6919206

[83] Wang Q Tang SB Song XB Deng TF Zhang TT Yin S. High-glucose concentrations change DNA methylation levels in human IVM oocytes. Hum Reprod. (2018) 33:474–81. 10.1093/humrep/dey006 29377995

[84] Nagashima JB El Assal R Songsasen N Demirci U. Evaluation of an ovary-on-a-chip in large mammalian models: species specificity and influence of follicle isolation status. J Tissue Eng Regen Med. (2018) 12:e1926–35. 10.1002/term.2623 29222841 PMC5906142

[85] Aziz AUR Fu M Deng J Geng C Luo Y Lin B. A microfluidic device for culturing an encapsulated ovarian follicle. Micromachines. (2017) 8:E335. 10.3390/mi8110335 30400524 PMC6190016

